# Cellular and soluble immune checkpoint signaling forms PD-L1 and PD-1 in renal tumor tissue and in blood

**DOI:** 10.1007/s00262-022-03166-9

**Published:** 2022-02-20

**Authors:** Corinna U. Keber, Marcus Derigs, Carolin Schultz, Moritz Wegner, Susanne Lingelbach, Viktoria Wischmann, Rainer Hofmann, Carsten Denkert, Axel Hegele, Jörg Hänze

**Affiliations:** 1 Clinic for Urology and Pediatric Urology, Faculty of Medicine, Philipps-University Marburg, Marburg, Germany; 2 Department of Pathology, Faculty of Medicine, Philipps-University Marburg, Marburg, Germany; 3 Urological Center Mittelhessen, DRK Hospital Biedenkopf, Biedenkopf, Germany; 4 Department of Vascular and Endovascular Surgery, Faculty of Medicine, University of Cologne and University Hospital Cologne, Cologne, Germany

**Keywords:** PD-L1, PD-1, sPD-L1, sPD-1, CRP, Tumor tissue, Blood serum, Whole blood RNA

## Abstract

Immune checkpoint blockade therapy is a treatment option of various metastatic cancer diseases including renal cell carcinoma (RCC). Approved antibody drugs target the co-inhibitory signaling of Programmed Cell Death Ligand-1 (PD-L1) and its receptor Programmed Cell Death-1 (PD-1). The combined evaluation of PD-L1 and PD-1 at the mRNA and protein levels in tumor tissue with differentiation of tumor and immune cells as well as of soluble forms (sPD-L1) and (sPD-1) in blood is of basic interest in assessing biomarker surrogates. Here, we demonstrate that PD-L1 determined as fraction of stained tumor cells (TPS-score) correlates with PD-L1-mRNA in tumor tissue, reflecting the predominant expression of PD-L1 in tumor cells. Conversely, PD-1 in immune cells of tumor tissue (IC-score) correlated with PD-1-mRNA tissue levels reflecting the typical PD-1 expression in immune cells. Of note, sPD-L1 in blood did not correlate with either the TPS-score of PD-L1 or with PD-L1-mRNA in tumor tissue. sPD-L1 released into the supernatant of cultured RCC cells closely followed the cellular PD-L1 expression as tested by interferon *γ* (IFNG) induction and siRNA knockdown of PD-L1. Further analysis in patients revealed that sPD-L1 significantly increased in blood following renal tumor resection. In addition, sPD-L1 correlated significantly with inflammation marker C-reactive protein (CRP) and with PD-L1 mRNA level in whole blood. These results indicate that the major source of sPD-L1 in blood may be peripheral blood cells and not primarily tumor tissue PD-L1.

**Supplementary Information:**

The online version contains supplementary material available at 10.1007/s00262-022-03166-9.

## Introduction

Programmed Cell Death (PD-1 or CD279) and Programmed Cell Death Ligand (PD-L1 or CD274) are co-inhibitory signaling components that act between antigen presenting cells and T-cells. PD-L1/PD-1 signaling suppresses major histocompatibility complex (MHC) and T-cell receptor (TCR) mediated antigen recognition [1] and thereby weakens foreign antigen recognition including neo-antigens in tumor cells. Thus, active PD-L1/PD-1 signaling disturbs cancer immune surveillance that arises from tumor infiltrating immune cells in the tumor microenvironment [2, 3]. The intercellular co-inhibitory action of PD-L1/PD-1 signaling can be successfully blocked by therapeutically administered antibodies that target PD-1 or PD-L1 [4–6]. Initially, these drug therapies were approved for melanoma and later for renal cell cancer [7, 8], but analog treatment of further malignancies, particularly, in advanced stages has also being used [9]. For RCC, the therapy response exerted by these drugs is significant; however, these drugs fail in a considerable portion of patients [10, 11]. As a primary drug target, PD-L1/PD-1 expression is investigated as a prognostic and predictive biomarker for disease outcome and therapy response toward PD-L1/PD-1 blockade [12–14]. Different parameters of PD-L1/PD-1 expression in tumor and immune cells as well as in blood can be measured in patients. PD-L1 and PD-1 expressed in tumor or immune cells are commonly assessed by immunohistochemistry. PD-L1-mRNA and PD-1-mRNA levels in tumor tissue more closely reflect transcriptional gene regulation. Soluble PD-L1 (sPD-L1) and soluble PD-1 (sPD-1) are detectable in peripheral blood but their cellular origin has not yet been clearly defined. sPD-L1 can be released by the shedding of membranous PD-L1 with ADAM10 and ADAM17 as relevant cleavage proteases [15–17]. In addition, alternative mRNA splice variants of the genes for PD-L1 [18, 19] and PD-1 [20, 21], which can be translated to secreted isoforms, have been identified. So far, exploiting PD-L1/PD-1 signaling as a biomarker for corresponding immune checkpoint blockade has not been conclusive in RCC patients [12, 17, 22] and the relationship between PD-L1 tumor expression and sPD-L1 in blood is unclear. The function of sPD-L1 and its value as a prognostic or predictive marker have been diversely described in the literature [15, 23].

In this study, the expression of PD-L1 and PD-1 in tumor tissue and their soluble forms sPD-L1 and sPD-1 in blood were evaluated in RCC patients. We aimed to verify possible associations between the parameters and to test for biomarker surrogates. In particular, PD-L1 and PD-1 were assessed by immunohistochemistry. PD-L1-mRNA and PD-1-mRNA, including markers of immune signaling, were measured in tissue homogenate. sPD-L1 and sPD-1 levels were measured before and after tumor resection. Significant correlations between some of the tissue parameters were found, but not between tissue and blood levels. Noteworthily, sPD-L1 in blood correlated with the inflammation marker C-reactive protein.

## Materials and methods

### Patients

**Fig. 1:**
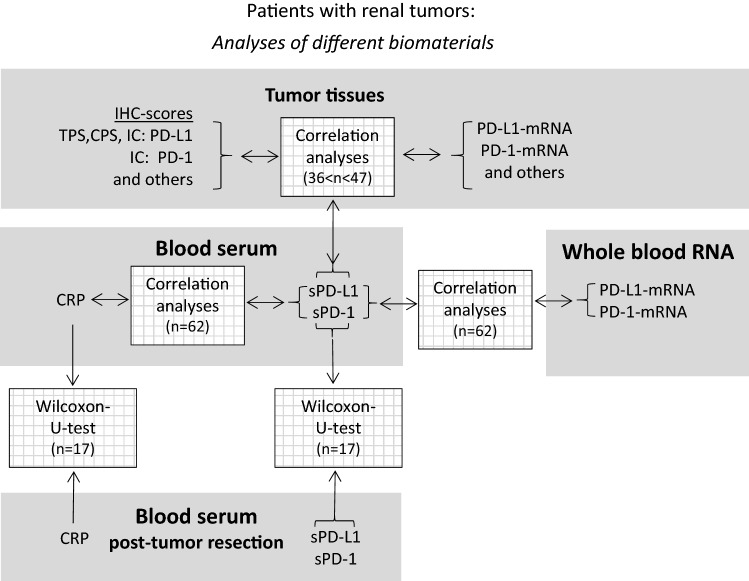
Study design. The analyzed biomaterials tumor tissue, blood serum and whole blood RNA from patients with renal tumors are highlighted in gray. The analyses that were performed between them are indicated by arrows in white shaded boxes

We prospectively analyzed renal tumor tissues and blood of patients undergoing renal tumor resection (radical nephrectomy or nephron sparing surgery). The patients were admitted from the commuting area of the hospital. The study was approved by the Philipps-University Marburg Ethics Committee (AZ 125/16), and written informed consent to participate in the study was obtained from all patients. An overview of the numbers of analyzed parameters along with statistics is summarized in a schematic diagram (Fig. 1). Characteristics of patients with clinicopathological evaluation of renal tumors are displayed in Table S1. The numbers of parameters vary since complete data sets were not retrieved from all patients’ samples. The actual number of parameters are indicated in Fig. 3 and supplementary Tables S2-S5.

### Immunohistochemistry and RNA extraction of tumor tissues

Formalin-fixated paraffin-embedded blocks of renal tumors were analyzed by immunohistochemistry. Heat-induced epitope retrieval was performed with EDTA for CD3 and with Trilogy for PD-L1, PD-1 and CD68. Staining was performed on a DAKO autostainer plus. After blocking endogenous peroxidase, sections were incubated for 45 min with rabbit monoclonal anti-PD-L1 antibody (1:1000; Cell Signaling #13,684, clone E1L3N), mouse monoclonal anti-CD68 antibody (1:100; Dako #M0876, clone PG-M1), mouse monoclonal anti-CD3 antibody (1:50; Dako #M7254, clone F7.2.38) and rabbit polyclonal anti-PD-1 antibody (1:150; Zytomed #516-18,662). Sections were washed and incubated with Dako REAL EnVision HRP Rabbit/Mouse polymer, which reacts with DAB-Chromogen, according to the manufacturer’s protocol. Different pathologic scores were determined [24–26], including tumor cell staining score (TPS-score), immune cell staining (IC-score) and a combined score (CPS-score) for PD-L1 and the immune cell staining scores (IC-score) for PD-1, CD3 and CD68, respectively. Total RNA was extracted from slices cut in line from the immunohistochemistry blocks (High Pure FFPE RNA Micro Kit, Roche Diagnostics GmbH, Germany).

### Processing of blood samples

sPD-L1 and sPD-1 were measured using commercial ELISA systems in blood serum (sPD-L1: R&D-Systems, Quantikine human B7-H1/PD-L1 DB7H10; sPD-1: R&D-Systems, DY1086 / Duo ancillary reagent kit 2 DY008). C-reactive protein (CRP) was determined within the clinical routine *testing.* RNA from whole blood was collected and extracted with the PAXgene system (PreAnalytiX, Qiagen: 763,134).

### Culturing and processing of cell lines

The cell lines were authenticated from the Leibniz Institute DSMZ-German Collection of Microorganisms and Cell Cultures, Germany, and are listed with the DSMZ-No. (CaKi1, ACC731; HepG2, ACC180; Jurkat, ACC282; THP-1, ACC-16). Cells were cultured in accordance with the recommended conditions in RPMI full growth medium supplemented with fetal bovine serum. The experiments were performed with tested mycoplasma-free cells (Minerva Biolabs GmbH, Berlin, Germany). Interleukin 2 (IL-2) (10 ng/ml), interleukin 6 (IL-6) (25 ng/ml), or interferon *γ* (IFNG) (10 ng/ml) from R&D Systems were added to the cells (24 h). RNA was extracted using the TriFast procedure (Peqlab, Erlangen, Germany). Protein was isolated with RIPA buffer (Cell signaling Technology Europe, Frankfurt a.M., Germany) supplemented with protease inhibitor cocktail (Sigma-Aldrich Chemie, GmbH, Munich, Germany) and phosphatase blocker (Phos-STOP, Roche Diagnostics GmbH, Mannheim, Germany). Extraction procedure was performed according to the manufacturers’ protocols. Cell supernatant was centrifuged for 10 min at 500 g and frozen at −20 °C until measurement of sPD-L1 and sPD-1 (see above). Transfection of double-stranded siRNA was performed with synthetic siRNAs sequences (Biomers GmbH, Germany) employing Lipofectamine RNAiMAX (Thermo Scientific Fisher). si-PD-L1 was compared to si-control sequence (supplementary Table S6). Cells were incubated with the respective siRNAs for 48 h and with IFNG in the last 24 h as indicated.

### Western blot

Protein samples (40 µg) were analyzed by sodium dodecyl sulfate polyacrylamide gel electrophoresis with subsequent electrotransfer to nitrocellulose membrane (Bio-Rad-System, Germany). The membranes were blocked in TRIS-buffered saline with 0.1% tween containing 5% dry milk and the primary antibodies were added and incubated at 4 °C for 24 h. The antibodies were as follows: PD-L1, host rabbit (#13,684, Cell signaling); PD-1, host goat (AF1086, R&D Systems); cytoplasmic *β*-actin, host mouse (#MAK6019 Linaris GmbH). Then, the respective secondary anti-host antibodies coupled with horseradish peroxidase were added for band detection with enhanced chemiluminescent luciferase kit (Thermo Scientific, Rockford, USA) by an imager system (Fluorchem IS-8900, Alpha Innotech, San Leandro, USA).

### cDNA synthesis and real-time RT-PCR

RNA (0.5 µg) was treated with DNAse I and cDNA synthesis was performed with random hexamer primers and M-MLV reverse transcriptase. The cDNA was submitted to SYBR green (ThermoScientific, UK) based RT-qPCR (IQ5, Biorad, Germany). Cycling conditions were as follows: 95 °C, 7.5 m followed by 40 cycles (95 °C, 15 s; 58 °C, 30 s; 72 °C, 30 s). Melting curve analysis was performed by a temperature increment of (0.5 °C, 10 s) from 60 °C up to 95 °C. Target mRNA levels are displayed as −∆Ct values (log 2-scale) normalized to TATA-binding protein (TBP)-mRNA as references. The primer sets (Biomers GmbH, Germany) were derived from sequence entries in GenBank and selected by Primer-Blast (NCBI National Center for Biotechnology Information). The corresponding sequences are listed (supplementary Table S7). For patients’ samples, the “PD-L1(s)” primer sets and for the cell line samples the “PD-L1” primer sets served for qPCR. The RT-PCR amplicons were characterized by DNA length and melting temperature profile and contaminations were excluded by appropriate controls (data not shown).

### Statistical analysis

Correlation analyses between TPS-score, IC-score, different mRNA target levels in tissue and whole blood as well as sPD-L1 and sPD-1 forms were performed by nonparametric Spearman test and linear regression (Pearson) analysis. Significant *p* values (*p* < 0.05) displayed in the supplementary Tables S2–S5 are highlighted in red. Comparison of data from pre- and post-tumor resection samples was performed by Wilcoxon test. The number is indicated in the figures and tables. The analyses were performed with MS Excel 2017 and Graphpad Prism 7.03.

## Results

### Immunohistochemical and mRNA analyses of PD-L1 and PD-1 in renal tumors

**Fig. 2:**
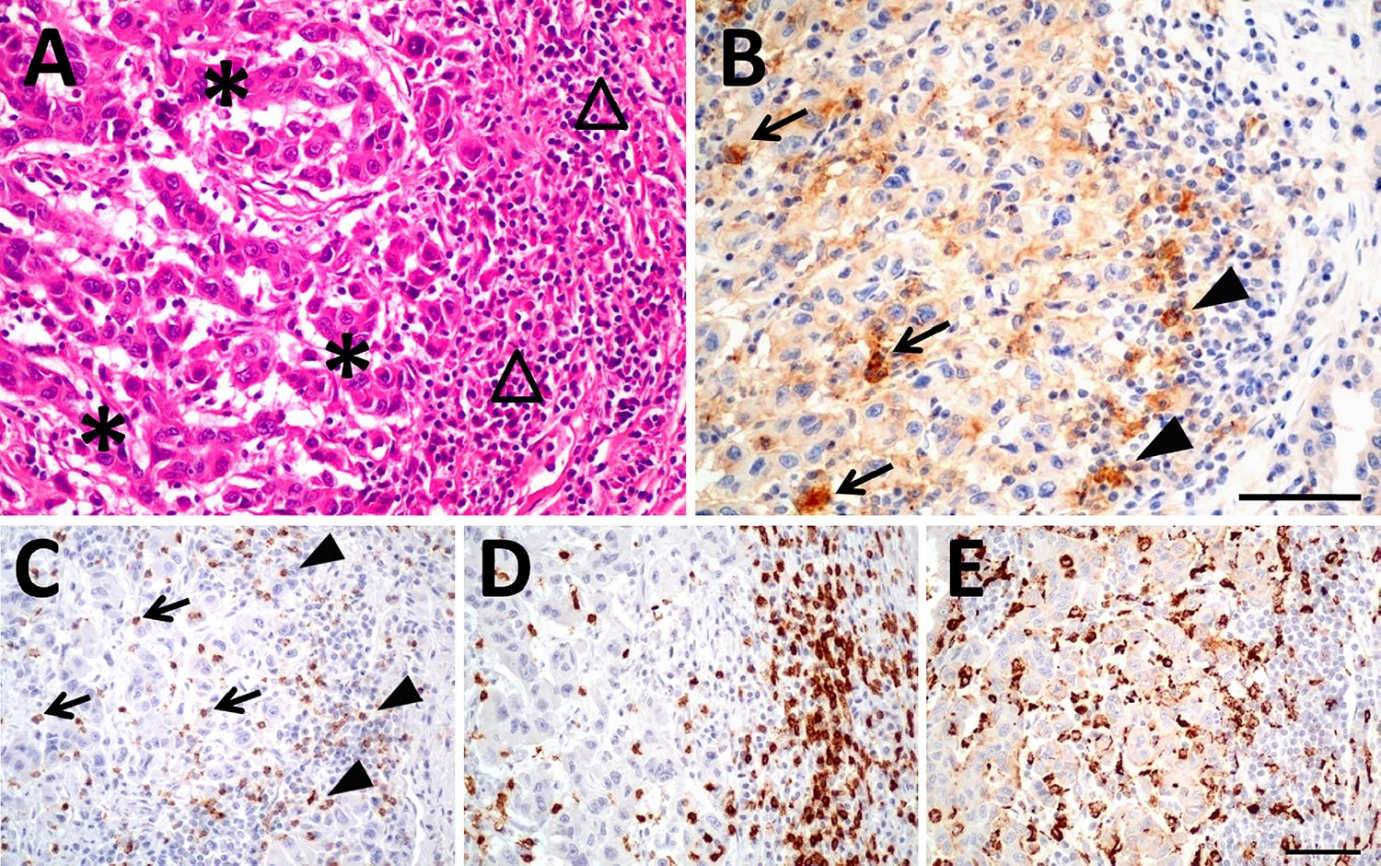
Histology and immunohistochemistry of a representative renal tumor. **A** HE staining of a poorly differentiated clear cell renal cell carcinoma showing tumor cells (*) and peritumoral lymphocytic infiltrate in the tumor stroma (triangle). **B** Immunostaining of PD-L1 demonstrates membranous staining of tumor cells as well as immune cells within the tumor (arrow) and in the adjacent stroma (arrowheads). **C** PD-1 is expressed in intratumoral (arrow) and stromal (arrowheads) lymphocytes. **D** CD 3 staining, labeling T-lymphocytes. **E** CD 68 staining, labeling macrophages. The scale bar indicates 100 µm

At first, we studied PD-L1 and PD-1 expression in renal tumor tissues. Immunohistochemically derived scores indicating the respective protein levels of PD-L1 and PD-1 in the tumor tissue served for correlation analyses with corresponding mRNA tissue levels and blood values of sPD-L1 and sPD-1 (see schematic diagram in Fig. 1). PD-L1 and PD-1 scores were determined along with markers of lymphocytes (CD3) and macrophages (CD68). We assessed the pathological scores that indicate the respective protein signals in immune and tumor cells. For markers expressed in immune cells such as PD-1, CD3, CD68 and PD-L1, the respective IC-scores were evaluated in tumor area (IC-tumor) and in stroma area (IC-stroma) of the tumor tissues. For PD-L1 that is predominantly expressed in tumor cells, we also determined the score in tumor cells (PD-L1-TPS) and a combined score for PD-L1 in tumor and immune cells (PD-L1-CPS). Exemplary figures of immunohistochemistry are displayed (Fig. 2). In the adjacent tumor tissue homogenates, we determined PD-L1-mRNA, PD-1-mRNA, CD3-mRNA and CD68-mRNA levels. Subsequent correlation analyses of the scores and the mRNA levels revealed certain traits of the targets (Table S2). In particular, (i) PD-L1-mRNA tissue level significantly correlated with PD-L1-TPS (*r* = 0.55; *p* = 0.00017) and with PD-L1-CPS (*r* = 0.38; *p *= 0.014) but not with PD-L1-IC scores. This reflects tumor cells as the major source of PD-L1 expression. (ii) PD-1-mRNA correlated with PD-1-IC in tumor (*r* = 0.33; *p* = 0.038) and PD-1-IC in stroma (*r* = 0.34; *p* = 0.04). Hence, immune cells represent the major source of PD-1 expression. (iii) In addition, CD3-mRNA in tumor tissue correlated with CD3 IC-tumor score (*R* = 0.44; *p* = 0.002) confirming T-cells as the major source of CD3-mRNA. CD68-mRNA did not correlate with CD68-IC-scores.

For delineation of PD-L1 expression with cytokine signaling, we also determined levels of JAK2-mRNA, CXCL10-mRNA and CXCR3-mRNA (Table S2 and S3). JAK2 is both mediator and target of IFNG signaling. CXCL10 and CXCR3 represent cytokine ligand/receptor end-targets of IFNG signaling [27–29]. Strikingly, PD-L1-mRNA correlated with JAK2-mRNA (*r* = 0.37; *p* = 0.011) possibly reflecting its dependence on IFNG signaling. For PD-1-mRNA, we observed several correlations with components enriched in immune cells (CD3-mRNA) (*r* = 0.37; *p* = 0.02) or indicating active immune response (JAK2-mRNA) (*r* = 0.73; *p* < 0.0001), CXCL10-mRNA (*r* = 0.62; *p* < 0.0001) and CXCR3-mRNA) (*r* = 0.53; *p* < 0.001).

Comparing the immunohistochemical scores, significant correlations were observed between PD-L1-TPS and CD3-IC of the tumor (*r* = 0.37; *p* = 0.015) and between PD-1-IC and CD3-IC (*r* = 0.43; *p* = 0.0032) (Table S4). PD-L1-IC-tumor correlated with PD-L1-IC-stroma (*r* = 0.54; *p* = 0.00018) and PD-1-IC-tumor correlated with PD-1-IC-stroma (*r* = 0.53; *p* = 0.00029) exhibiting some similarities between both compartments.

### sPD-L1 and sPD-1 in blood of patients

sPD-L1 and sPD-1 were measured in patients’ blood samples, and both sPD-L1 and sPD-1 were detectable at heterogeneous levels in all blood samples. Importantly, sPD-L1 did not correlate with PD-L1-mRNA levels in tumor tissue, nor with TPS- and IC-scores of PD-L1. In addition, sPD-1 did not correlate with PD-1-mRNA in tumor tissue nor with IC-scores of PD-1 and no correlation was found between sPD-1 and sPD-L1 (Table S5).

**Fig. 3:**
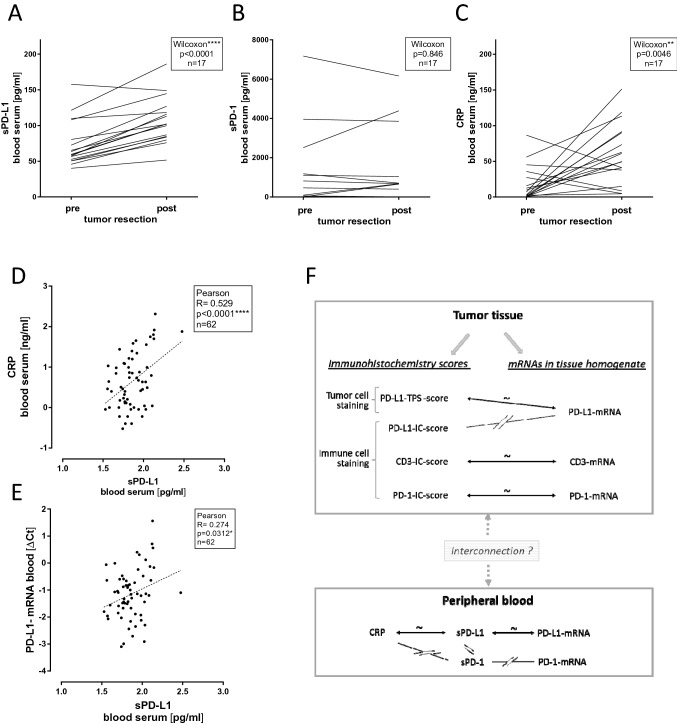
Analysis of peripherally collected venous blood. **A**, **B**, **C** Blood values of patients collected before (pre) and after (post) tumor resection for sPD-L1 (**A**), sPD-1 (**B**), CRP (**C**) (time between collection of pre- and post-samples: median 15 days; min/max 8/57 days). For Wilcoxon parameters see insets. **D**, **E** Linear regression analyses between sPD-L1 and CRP values and between sPD-L1 and PD-L1-mRNA determined in whole blood RNA. Here, all samples were collected from patients before tumor resection. For linear regression parameters see insets [total number: *n* = 62; male: *n* = 37 female: *n* = 25; age (years: mean ± SD): 65.4 ± 11.6; min/max 25/83]. **F** Schematic diagram summarizing the observed associations between mRNA and protein markers in tumor tissue and in peripheral blood compartments of patients. Lines with tilde and arrows indicate significant correlation. Interrupted lines indicate no significant correlation. The interconnection between the parameters of tumor tissue and peripheral blood is not clear

Next, we compared sPD-L1 and sPD-1 in the blood of patients before and after tumor resection. Notably, the sPD-L1 values increased significantly in blood collected after tumor resection compared to sPD-L1 levels before tumor resection (sPD-L1 [pg/ml]; mean ± SD; pre: 76 ± 32; post: 107 ± 32; *p* < 0.0001) (Fig. 3A). In contrast, there was no difference in the sPD-1 levels before and after tumor resection (sPD-1 [pg/ml]; mean ± SD; pre: 1027 ± 1922; post: 1097 ± 1849; pg/ml; *p* = 0.8457) (Fig. 3B). As expected, the inflammation marker CRP increased in the blood samples post-tumor resection (CRP [ng/ml]; mean ± SD; pre: 18.1 ± 24.6; post: 59.6 ± 42.5; *p* = 0.0046) (Fig. 3C), reflecting the surgical insult [30]. In addition, CRP correlated significantly with sPD-L1 levels in blood (Fig. 3D). Furthermore, we observed significant correlation of sPD-L1 with PD-L1-mRNA levels determined in whole blood (Fig. 3E). The striking relationships of parameters in tumor tissue and blood are outlined in a hypothetical schematic diagram (Fig. 3F).

### PD-L1 and PD-1 expression in cell lines

**Fig. 4:**
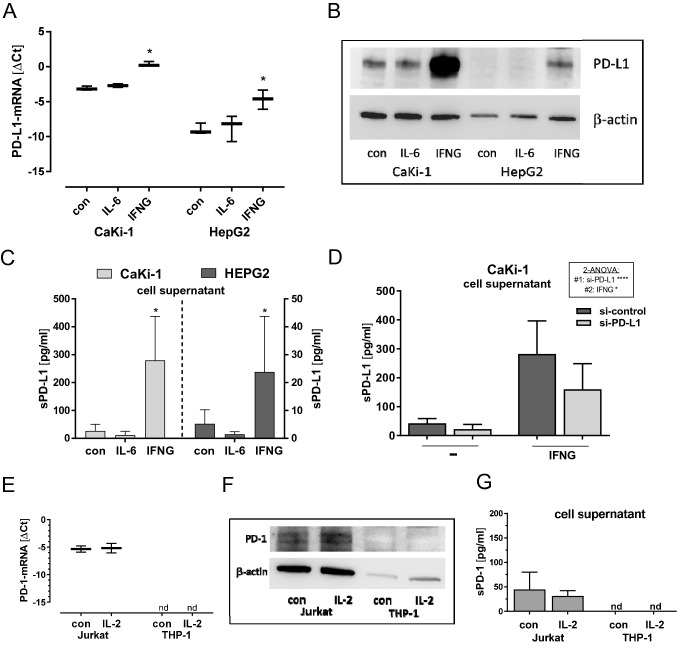
*PD-L1 and PD-1 expression in cell lines.* **A**, **B**, **C** PD-L1 in CaKi-1 and HepG2 cells that were treated with IL-6 (10 ng/ml) or IFNG (10 ng/ml). **A** PD-L1-mRNA (Mann–Whitney-Test *p < 0.05, *n* = 3). **B** Western blot of PD-L1 (50kd) and *β*-Actin (43kd). **C** sPD-L1 in cell supernatant (Mann–Whitney-Test *p < 0.05, *n* = 3). **D** CaKi-1 cells grouped by treatment with IFNG and si-PD-L1 for knockdown of PD-L1 (2-way ANOVA: si-PD-L1: *f* = 34.86, *p* =  < 0.0001; IFNG: *f* = 4.97, *p* = 0.0154; Interaction: *f* = 2.54, *p* = 0.0769). **E**, **F**, **G**: PD-1 in Jurkat and THP-1 cells that were treated with IL-2 (10 ng/ml). **E** PD-1-mRNA (*n* = 3). **F** Western blot of PD-1 (50kd) and *β*-actin (43kd). **G** sPD-1 in cell supernatant

To characterize the regulation of PD-L1 and PD-1 along with sPD-L1 and sPD-1, we investigated cancer (CaKi-1, HEP-G2) and immune cell lines (Jurkat, THP-1). CaKi-1 and HEP-G2 cells displayed typical induction of PD-L1 by IFNG paralleled by release of sPD-L1 into the cell supernatant. In contrast to IFNG, IL-6 did not induce PD-L1 neither in CaKi-1 cells nor in HEP-G2 cells that were employed as principal IL-6 responsive cell line [31] (Fig. 4A–C). Silencing of sPD-L1 by siRNA in CaKi-1 cells reduced sPD-L1 level in supernatant confirming a direct link between cellular PD-L1 expression and the release of sPD-L1 into the supernatant (Fig. 4D). Considering PD-1 expression, it turned out that Jurkat cells displayed detectable levels of PD-1-mRNA whereas THP-1 did not. Accordingly, Jurkat cells revealed immune-reactive PD-1 bands in Western blot that were not detectable in THP-1 cells. IL-2, a possible inducer of PD-1 [32], had no considerable effect on PD-1 levels (Fig. 4E, F). In supernatants of Jurkat cells, sPD-1 levels were slightly above detection threshold (Fig. 4G).

## Discussion

We determined PD-L1/PD-1 in resected renal tumor tissues along with the soluble forms sPD-L1/sPD-1 in patient’s blood. Subsequent data analyses tested for related biomarker surrogates. The parameters comprised (i) immunohistochemical scores for PD-L1 and PD-1 in immune and tumor cells, (ii) PD-L1-mRNA and PD-1-mRNA concentrations in the adjacent tissue slides, (iii) sPD-L1 and sPD-1 in blood serum, (iv) PD-L1-mRNA and PD-1-mRNA in whole blood RNA and (v) comparison of sPD-L1 and sPD-1 in blood before and after tumor resection.

### IHC-scores of PD-L1 and PD-1 versus mRNAs in tumor tissues

Comparing immunohistochemistry and mRNA data (Table S2), we found correlations between PD-L1-TPS and PD-L1-mRNA and between PD-1-IC-score and PD-1-mRNA in the respective adjacent tissue layers. This observation reflects predominant congruent expression of PD-L1-mRNA and PD-L1 protein in tumor cells and between PD-1-mRNA and PD-1 protein in immune cells [33]. For PD-1, we determined correlation of mRNA level with several immune cell specific markers (CD3, CD68) including the cytokine signaling markers CXCL10 and CXCR3 that are engaged in active immune cell responses [27–29, 34].

### PD-L1 expression in tumor tissue and cells in relation to sPD-L1 in blood

We observed that PD-L1 protein and mRNA expression in tumor tissue was not associated with sPD-L1 levels in circulating blood (Table S5). Likewise, in a study of hepatocellular carcinoma (HCC), no link between immunohistochemical staining and sPD-L1 was detected [35]. These observations are not implicitly expected since both RCC and HCC cell lines release sPD-L1 into cell supernatant (Fig. 4B, C). Of note, it is speculated that the amounts of sPD-L1 in blood versus PD-L1 in tumor tissue may be reciprocally interconnected [15]. In particular, active metalloproteases ADAM10/17 can release sPD-L1 from tumor tissue. Thereby, immunohistochemical detectable PD-L1 in tissue attenuates whereas sPD-L1 in blood increases. Our experimental tools cannot directly track this process. We found neither positive nor negative correlations of sPD-L1 with PD-L1 tissue expression when considering protein and mRNA levels, or their ratios.

### sPD-L1 in relation to inflammation marker CRP and PD-L1-mRNA in blood

The increased levels of sPD-L1 after tumor resection suggest a primarily non-tumor derived origin of sPD-L1 in blood (Fig. 3A). In parallel, CRP was elevated in the post- versus pre-tumor resection samples (Fig. 3C), an anticipated effect due to tissue damage and wound healing processes after the surgical insult [30]. Corresponding analysis revealed that sPD-L1 also correlated with CRP in the pre-tumor resection samples (Fig. 3D). This trait of sPD-L1 has been described in pancreatic cancer patients [35] and HCC patients [36] where dichotomized patients groups with high and low CRP values were compared. Additionally, in patients with pancreatic cancer, an association between sPD-L1 and sPD-1 was revealed [35] that was not noticed in HCC patients [36] and not in our study.

Of note, the tumor volume may particularly add to the pre-surgery levels of sPD-L1 levels in blood and may overlap with the inflammatory conditions regulating sPD-L1.

Another point is that we noticed correlation of sPD-L1 in serum with PD-L1 mRNA in whole blood (Fig. 3E). This observation supports peripheral blood cells as a relevant source for sPD-L1. In line, activated mature dendritic cells isolated from peripheral blood mononuclear cells (PBMNs) were shown to release sPD-L1 [16]. Furthermore, transcriptomic RNAseq analysis identified PD-L1 transcripts in sorted peripheral blood cells from healthy persons that were accessible from the GTEx database [19]. In particular, polymorphonuclear neutrophil granulocytes (PMNs), T-lymphocytes, B-lymphocytes and monocytes exhibited considerable levels of PD-L1-mRNAs expressed as full length along with truncated splice variants.

IL-6 signaling represents a major pathway for induction of acute phase proteins such as CRP in hepatocytes during inflammation [31, 37]. Since sPD-L1 correlated with CRP, we tested for induction of PD-L1 and sPD-L1 through IL-6. Analysis of cultured RCC and HCC cell lines could not demonstrate any effects of IL-6 on PD-L1 and sPD-L1 (Fig. 4). Speculatively, IL-6 may affect sPD-L1 levels by course of events in blood. Possibly, IL-6 triggered inhibition of apoptosis of neutrophil granulocytes [38] may favor sPD-L1 enrichment in blood.

For sPD-1, we confirmed PD-1 mRNA expression in the T-cell derived Jurkat cell line [39, 40] and additionally detected soluble sPD-1 in the Jurkat cell supernatant. In contrast to sPD-L1, we did not observe alterations of sPD-1 in response to renal tumor resection.

In summary, soluble PD-L1 in blood correlated with inflammation marker C-reactive protein and with PD-L1-mRNA level in whole blood. Of note, sPD-L1 was not associated with PD-L1 expression in renal tumors.

### Supplementary Information

Below is the link to the electronic supplementary material.

Supplementary file1 (PDF 533 KB)

Supplementary file2 (PDF 412 KB)

Supplementary file3 (PDF 407 KB)

Supplementary file4 (PDF 416 KB)

Supplementary file5 (PDF 390 KB)

Supplementary file6 (PDF 372 KB)

Supplementary file7 (PDF 385 KB)
