# Supplementary material for: Cellular and soluble immune checkpoint signaling forms PD-L1 and PD-1 in renal tumor tissue and in blood
Source: Cancer Immunol Immunother. 2022 Feb 20;71(10):2381–9. doi: 10.1007/s00262-022-03166-9 (PMC9463294; doi:10.1007/s00262-022-03166-9)
Supplement: Supplementary file 1 — Supplementary file1 (PDF 533 KB) [file 262_2022_3166_MOESM1_ESM.pdf]

Table S1:

**Basic characteristics of patients**	**Tissue sample analyses**	**Blood sample analyses**
age	*years*	*years*
Mean $\pm$ SD	65,2 $\pm$ 12,6	65,4 $\pm$ 11,6
Min / Max	26 / 83	25 / 82
Sex	*Number (%)*	*Number (%)*
Male	26 (55%)	37 (59,7%)
Female	21 (45%)	25 (40,3%)
Pathologic diagnosis of kidney tumors	*Number (%)*	*Number (%)*
Clear cell RCC	27 (57%)	31 (50%)
Papillary RCC	7 (15%)	8 (12,9%)
Chromophobe RCC	2 (4,3%)	2 (3,2%)
Urothelial carcinoma	7 (15%)	9 (14,5%)
Angiomyolipoma	3(6,4%)	4(6,5%)
Liposarcoma	1 (2,1%)	1 (1,6 %)
n.a.	0 (0%)	7 (11,3%)
Total	*47 (100%)*	*62 (100%)*
