# Supplementary material for: Cellular and soluble immune checkpoint signaling forms PD-L1 and PD-1 in renal tumor tissue and in blood
Source: Cancer Immunol Immunother. 2022 Feb 20;71(10):2381–9. doi: 10.1007/s00262-022-03166-9 (PMC9463294; doi:10.1007/s00262-022-03166-9)
Supplement: Supplementary file 2 — Supplementary file2 (PDF 412 KB) [file 262_2022_3166_MOESM2_ESM.pdf]

Table S2:

Correlations of IHC-scores and tissue mRNAs

PD-L1 TPS-%				PD-L1 CPS			
**spearman**	**r**	**p**	**n**	**spearman**	**r**	**p**	**n**
**PD-L1-mRNA**	0,55	1,7E-04	42	**PD-L1-mRNA**	0,38	1,4E-02	42
**PD-1-mRNA**	0,35	3,7E-02	36	**PD-1-mRNA**	0,24	1,6E-01	36
**CD3-mRNA**	0,37	1,6E-02	43	**CD3-mRNA**	0,34	2,7E-02	43
**CD68-mRNA**	0,11	4,8E-01	43	**CD68-mRNA**	0,20	1,9E-01	43
**Jak2-mRNA**	0,34	2,6E-02	43	**Jak2-mRNA**	0,15	3,2E-01	43
**CXCL10-mRNA**	0,11	4,9E-01	43	**CXCL10-mRNA**	0,09	5,8E-01	43
**CXCR3-mRNA**	0,10	5,4E-01	43	**CXCR3-mRNA**	0,12	4,3E-01	43

PD-L1 IC-Tumor%				PD-L1 IC-Stroma%			
**spearman**	**r**	**p**	**n**	**spearman**	**r**	**p**	**n**
**PD-L1-mRNA**	0,28	7,5E-02	42	**PD-L1-mRNA**	0,07	6,5E-01	42
**PD-1-mRNA**	0,17	3,3E-01	36	**PD-1-mRNA**	0,02	8,9E-01	36
**CD3-mRNA**	0,26	9,9E-02	43	**CD3-mRNA**	0,17	2,8E-01	43
**CD68-mRNA**	0,19	2,3E-01	43	**CD68-mRNA**	0,13	4,1E-01	43
**Jak2-mRNA**	0,10	5,2E-01	43	**Jak2-mRNA**	-0,06	7,0E-01	43
**CXCL10-mRNA**	0,05	7,3E-01	43	**CXCL10-mRNA**	0,07	6,5E-01	43
**CXCR3-mRNA**	0,20	1,9E-01	43	**CXCR3-mRNA**	0,15	3,4E-01	43

PD-1 IC-Tumor%				PD-1 IC-Stroma%			
**spearman**	**r**	**p**	**n**	**spearman**	**r**	**p**	**n**
**PD-L1-mRNA**	0,07	6,3E-01	46	**PD-L1-mRNA**	-0,08	6,0E-01	42
**PD-1-mRNA**	0,33	3,8E-02	39	**PD-1-mRNA**	0,34	4,0E-02	36
**CD3-mRNA**	0,60	7,1E-06	47	**CD3-mRNA**	0,57	5,9E-05	43
**CD68-mRNA**	0,39	6,8E-03	47	**CD68-mRNA**	0,25	1,1E-01	43
**Jak2-mRNA**	0,22	1,4E-01	47	**Jak2-mRNA**	0,19	2,3E-01	43
**CXCL10-mRNA**	0,56	5,0E-05	47	**CXCL10-mRNA**	0,37	1,4E-02	43
**CXCR3-mRNA**	0,53	1,3E-04	47	**CXCR3-mRNA**	0,46	1,9E-03	43

CD3 IC-Tumor%				CD3 IC-Stroma%			
**spearman**	**r**	**p**	**n**	**spearman**	**r**	**p**	**n**
**PD-L1-mRNA**	0,09	5,7E-01	45	**PD-L1-mRNA**	-0,10	5,2E-01	41
**PD-1-mRNA**	0,13	4,3E-01	38	**PD-1-mRNA**	0,34	4,5E-02	35
**CD3-mRNA**	0,44	2,0E-03	46	**CD3-mRNA**	0,30	5,3E-02	42
**CD68-mRNA**	0,11	4,7E-01	46	**CD68-mRNA**	0,07	6,7E-01	42
**Jak2-mRNA**	0,10	5,1E-01	46	**Jak2-mRNA**	0,24	1,2E-01	42
**CXCL10-mRNA**	0,19	2,1E-01	46	**CXCL10-mRNA**	0,13	4,1E-01	42
**CXCR3-mRNA**	0,24	1,2E-01	46	**CXCR3-mRNA**	0,21	1,9E-01	42

CD68 IC-Tumor%				CD68 IC-Stroma%			
**spearman**	**r**	**p**	**n**	**spearman**	**r**	**p**	**n**
**PD-L1-mRNA**	-0,12	4,5E-01	46	**PD-L1-mRNA**	0,04	8,1E-01	42
**PD-1-mRNA**	0,27	9,1E-02	39	**PD-1-mRNA**	0,10	5,8E-01	36
**CD3-mRNA**	0,19	2,0E-01	47	**CD3-mRNA**	0,11	4,6E-01	43
**CD68-mRNA**	0,15	3,0E-01	47	**CD68-mRNA**	-0,01	9,6E-01	43
**Jak2-mRNA**	0,14	3,5E-01	47	**Jak2-mRNA**	0,26	9,1E-02	43
**CXCL10-mRNA**	0,02	9,2E-01	47	**CXCL10-mRNA**	0,19	2,2E-01	43
**CXCR3-mRNA**	0,18	2,3E-01	47	**CXCR3-mRNA**	-0,02	8,7E-01	43

Note: p-values<0.05 are highlighted in red
