# Supplementary material for: Cellular and soluble immune checkpoint signaling forms PD-L1 and PD-1 in renal tumor tissue and in blood
Source: Cancer Immunol Immunother. 2022 Feb 20;71(10):2381–9. doi: 10.1007/s00262-022-03166-9 (PMC9463294; doi:10.1007/s00262-022-03166-9)
Supplement: Supplementary file 3 — Supplementary file3 (PDF 407 KB) [file 262_2022_3166_MOESM3_ESM.pdf]

Table S3:			
**Correlations of tissue mRNAs**			
	**PD-L1-mRNA**		
**spearman**	**r**	**p**	**n**
**PD-L1-mRNA**			
**PD-1-mRNA**	0,13	4,5E-01	38
**CD3-mRNA**	0,01	9,7E-01	46
**CD68-mRNA**	0,16	3,0E-01	46
**Jak2-mRNA**	0,37	1,1E-02	46
**CXCL10-mRNA**	0,21	1,7E-01	46
**CXCR3-mRNA**	0,05	7,4E-01	46
	**PD-1-mRNA**		
**spearman**	**r**	**p**	**n**
**PD-L1-mRNA**	0,13	4,5E-01	38
**PD-1-mRNA**			
**CD3-mRNA**	0,60	5,1E-05	39
**CD68-mRNA**	0,55	3,3E-04	39
**Jak2-mRNA**	0,66	5,1E-06	39
**CXCL10-mRNA**	0,56	2,0E-04	39
**CXCR3-mRNA**	0,63	1,7E-05	39

Note: p-values<0.05 are highlighted in red
