# Supplementary material for: Cellular and soluble immune checkpoint signaling forms PD-L1 and PD-1 in renal tumor tissue and in blood
Source: Cancer Immunol Immunother. 2022 Feb 20;71(10):2381–9. doi: 10.1007/s00262-022-03166-9 (PMC9463294; doi:10.1007/s00262-022-03166-9)
Supplement: Supplementary file 4 — Supplementary file4 (PDF 416 KB) [file 262_2022_3166_MOESM4_ESM.pdf]

Table S4:
Correlations of IHC-scores

		PD-L1 TPS-%		
spearman		r	p	n
PD-L1	TPS-%			
	CPS	0,66	1,8E-06	43
	IC-Tumor%	0,36	1,8E-02	43
	IC-Stroma%	0,20	1,9E-01	43
PD-1	IC-Tumor%	0,11	5,0E-01	43
	IC-Stroma%	0,19	2,3E-01	43
CD3	IC-Tumor%	0,37	1,5E-02	42
	IC-Stroma%	0,11	5,0E-01	42
CD68	IC-Tumor%	0,14	3,7E-01	43
	IC-Stroma%	0,08	6,1E-01	43

		PD-L1 CPS		
spearman		r	p	n
PD-L1	TPS-%	0,66	1,8E-06	43
	CPS			
	IC-Tumor%	0,57	7,3E-05	43
	IC-Stroma%	0,65	3,0E-06	43
PD-1	IC-Tumor%	0,06	7,0E-01	43
	IC-Stroma%	0,44	3,4E-03	43
CD3	IC-Tumor%	0,29	6,3E-02	42
	IC-Stroma%	0,20	1,9E-01	42
CD68	IC-Tumor%	0,08	5,9E-01	43
	IC-Stroma%	0,28	7,2E-02	43

		PD-L1 IC-Tumor%		
spearman		r	p	n
PD-L1	TPS-%	0,36	1,8E-02	43
	CPS	0,57	7,3E-05	43
	IC-Tumor%			
	IC-Stroma%	0,54	1,8E-04	43
PD-1	IC-Tumor%	0,28	6,4E-02	43
	IC-Stroma%	0,33	3,1E-02	43
CD3	IC-Tumor%	0,05	7,5E-01	42
	IC-Stroma%	0,19	2,3E-01	42
CD68	IC-Tumor%	-0,03	8,4E-01	43
	IC-Stroma%	0,29	6,1E-02	43

		PD-L1 IC-Stroma%		
spearman		r	p	n
PD-L1	TPS-%	0,20	1,9E-01	43
	CPS	0,65	3,0E-06	43
	IC-Tumor%	0,54	1,8E-04	43
	IC-Stroma%			
PD-1	IC-Tumor%	0,02	8,8E-01	43
	IC-Stroma%	0,48	9,8E-04	43
CD3	IC-Tumor%	-0,01	9,5E-01	42
	IC-Stroma%	0,30	5,0E-02	42
CD68	IC-Tumor%	0,04	8,1E-01	43
	IC-Stroma%	0,16	3,0E-01	43

		PD-1 IC-Tumor%		
spearman		r	p	n
PD-L1	TPS-%	0,11	5,0E-01	43
	CPS	0,06	7,0E-01	43
	IC-Tumor%	0,28	6,4E-02	43
	IC-Stroma%	0,02	8,8E-01	43
PD-1	IC-Tumor%			
	IC-Stroma%	0,53	2,9E-04	43
CD3	IC-Tumor%	0,43	3,2E-03	46
	IC-Stroma%	0,10	5,4E-01	42
CD68	IC-Tumor%	0,15	3,2E-01	47
	IC-Stroma%	0,18	2,6E-01	43

		PD-1 IC-Stroma%		
spearman		r	p	n
PD-L1	TPS-%	0,19	2,3E-01	43
	CPS	0,44	3,4E-03	43
	IC-Tumor%	0,33	3,1E-02	43
	IC-Stroma%	0,48	9,8E-04	43
PD-1	IC-Tumor%	0,53	2,9E-04	43
	IC-Stroma%			
CD3	IC-Tumor%	0,23	1,5E-01	42
	IC-Stroma%	0,47	1,6E-03	42
CD68	IC-Tumor%	0,15	3,4E-01	43
	IC-Stroma%	0,29	5,9E-02	43

		CD3 IC-Tumor%		
spearman		r	p	n
PD-L1	TPS-%	0,37	1,5E-02	42
	CPS	0,29	6,3E-02	42
	IC-Tumor%	0,05	7,5E-01	42
	IC-Stroma%	-0,01	9,5E-01	42
PD-1	IC-Tumor%	0,43	3,2E-03	46
	IC-Stroma%	0,23	1,5E-01	42
CD3	IC-Tumor%			
	IC-Stroma%	-0,04	8,0E-01	41
CD68	IC-Tumor%	0,35	1,6E-02	46
	IC-Stroma%	-0,12	4,6E-01	42

		CD3 IC-Stroma%		
spearman		r	p	n
PD-L1	TPS-%	0,11	5,0E-01	42
	CPS	0,20	1,9E-01	42
	IC-Tumor%	0,19	2,3E-01	42
	IC-Stroma%	0,30	5,0E-02	42
PD-1	IC-Tumor%	0,10	5,4E-01	42
	IC-Stroma%	0,47	1,6E-03	42
CD3	IC-Tumor%	-0,04	8,0E-01	41
	IC-Stroma%			
CD68	IC-Tumor%	0,27	7,8E-02	42
	IC-Stroma%	0,24	1,3E-01	42

		CD68 IC-Tumor%		
spearman		r	p	n
PD-L1	TPS-%	0,14	3,7E-01	43
	CPS	0,08	5,9E-01	43
	IC-Tumor%	-0,03	8,4E-01	43
	IC-Stroma%	0,04	8,1E-01	43
PD-1	IC-Tumor%	0,15	3,2E-01	47
	IC-Stroma%	0,15	3,4E-01	43
CD3	IC-Tumor%	0,35	1,6E-02	46
	IC-Stroma%	0,27	7,8E-02	42
CD68	IC-Tumor%			
	IC-Stroma%	-0,23	1,4E-01	43

		CD68 IC-Stroma%		
spearman		r	p	n
PD-L1	TPS-%	0,08	6,1E-01	43
	CPS	0,28	7,2E-02	43
	IC-Tumor%	0,29	6,1E-02	43
	IC-Stroma%	0,16	3,0E-01	43
PD-1	IC-Tumor%	0,18	2,6E-01	43
	IC-Stroma%	0,29	5,9E-02	43
CD3	IC-Tumor%	-0,12	4,6E-01	42
	IC-Stroma%	0,24	1,3E-01	42
CD68	IC-Tumor%	-0,23	1,4E-01	43
	IC-Stroma%			

Note: p-values<0.05 are highlighted in red
