# Supplementary material for: Cellular and soluble immune checkpoint signaling forms PD-L1 and PD-1 in renal tumor tissue and in blood
Source: Cancer Immunol Immunother. 2022 Feb 20;71(10):2381–9. doi: 10.1007/s00262-022-03166-9 (PMC9463294; doi:10.1007/s00262-022-03166-9)
Supplement: Supplementary file 5 — Supplementary file5 (PDF 390 KB) [file 262_2022_3166_MOESM5_ESM.pdf]

Table S5:

Correlations of sPD-L1 and sPD-1 in blood versus tumor tissue IHC and mRNAs

		sPD-L1		
	**spearman**	**r**	**p**	**n**
	**sPD-L1**			
	**sPD-1**	-0,01	9,4E-01	46
**PD-L1**	**TPS-Score%**	0,13	4,1E-01	42
	**CPS-Scor**	-0,08	6,2E-01	42
	**IC-Tumor%**	-0,08	6,2E-01	42
	**IC-Stroma%**	-0,22	1,6E-01	42
**PD-L1-mRNA**		0,20	1,8E-01	45

		sPD-1		
	**spearman**	**r**	**p**	**n**
	**sPD-L1**	-0,01	9,4E-01	46
	**sPD-1**			
**PD-1**	**IC-Tumor%**	0,01	9,6E-01	46
	**IC-Stroma%**	0,06	7,0E-01	42
**PD-1-mRNA**		-0,16	3,4E-01	38
