# Supplementary material for: Cellular and soluble immune checkpoint signaling forms PD-L1 and PD-1 in renal tumor tissue and in blood
Source: Cancer Immunol Immunother. 2022 Feb 20;71(10):2381–9. doi: 10.1007/s00262-022-03166-9 (PMC9463294; doi:10.1007/s00262-022-03166-9)
Supplement: Supplementary file 6 — Supplementary file6 (PDF 372 KB) [file 262_2022_3166_MOESM6_ESM.pdf]

**Table S6:**		
**si-RNA sequences**		
si-PD-L1+	CCUACUGGCAUUUGCUGAACGCAUU	
si-PD-L1-	AAUGCGUUCAGCAAAUGCCAGUAGG	
si-control+	UAAGGCUAUGAAGAGAUAC	
si-control-	GUAUCUCUUCAUAGCCUUA	
