# Supplementary material for: Cellular and soluble immune checkpoint signaling forms PD-L1 and PD-1 in renal tumor tissue and in blood
Source: Cancer Immunol Immunother. 2022 Feb 20;71(10):2381–9. doi: 10.1007/s00262-022-03166-9 (PMC9463294; doi:10.1007/s00262-022-03166-9)
Supplement: Supplementary file 7 — Supplementary file7 (PDF 385 KB) [file 262_2022_3166_MOESM7_ESM.pdf]

Table S7:

Primer sequences for RT-qPCR	
PD-L1+	GCGAATTACTGTGAAAGTCAATGCC
PD-L1-	TGGTCACATTGAAAAGCTTCTCCTC
PD-L1(s)+	TGTGACCAGCACACTGAGAATCA
PD-L1(s)-	TGGAGGATGTGCCAGAGGTAGT
PD-1+	GGCCGCACGAGGGACAATAG
PD-1-	AGGAAAGACAATGGTGGCATACTCC
PD-1(s)+	CCGGCCAGTTCCAAACCCTG
PD-1(s)-	TGCGCCTGGCTCCTATTGTCC
CD3e+	GCTACCCCAGAGGAAGCAAACCA
CD3e-	ACAGGCTTGGCCTTGGCCTT
CD68+	GTGCCCATCCCCACCTGCTT
CD68-	GAAGCTCTGCCCCAGGGGTG
JAK2+	GCCCTGGGGTTTTCTGGTGC
JAK2-	CCGGCACATCTCCACACTCCC
CXCL10+	ACGCTGTACCTGCATCAGCA
CXCL10-	TTCTTGATGGCCTTCGATTCTGGA
CXCR3+	GCCTGCATCAGCTTTGACCGC
CXCR3-	TTGAGGCGCTCGTCGTGGTG
TBP+	TCATGAGGATAAGAGAGCCACGAAC
TBP-	TAGGAAACTTCACATCACAGCTCCC
